# The role of bone marrow on the mechanical properties of trabecular bone: a systematic review

**DOI:** 10.1186/s12938-022-01051-1

**Published:** 2022-11-23

**Authors:** Fangxing Wang, Florian Metzner, Georg Osterhoff, Leyu Zheng, Stefan Schleifenbaum

**Affiliations:** 1 ZESBO - Center for Research on Musculoskeletal Systems, Department of Orthopedic Surgery, Traumatology and Plastic Surgery, Leipzig University, Semmelweisstraße 14, 04103 Leipzig, Germany; 2 Department of Orthopedic Surgery, Traumatology and Plastic Surgery, Leipzig University, Liebigstraße 20 Haus 4, 04103 Leipzig, Germany

**Keywords:** Bone, Bone marrow, Trabecular bone, Mechanical properties, Mechanical testing

## Abstract

**Background:**

Accurate evaluation of the mechanical properties of trabecular bone is important, in which the internal bone marrow plays an important role. The aim of this systematic review is to investigate the roles of bone marrow on the mechanical properties of trabecular bone to better support clinical work and laboratory research.

**Methods:**

A systematic review of the literature published up to June 2022 regarding the role of bone marrow on the mechanical properties of trabecular bone was performed, using PubMed and Web of Science databases. The journal language was limited to English. A total of 431 articles were selected from PubMed (*n* = 186), Web of Science (*n* = 244) databases, and other sources (*n* = 1).

**Results:**

After checking, 38 articles were finally included in this study. Among them, 27 articles discussed the subject regarding the hydraulic stiffening of trabecular bone due to the presence of bone marrow. Nine of them investigated the effects of bone marrow on compression tests with different settings, i.e., in vitro experiments under unconfined and confined conditions, and computer model simulations. Relatively few controlled studies reported the influence of bone marrow on the shear properties of trabecular bone.

**Conclusion:**

Bone marrow plays a non-neglectable role in the mechanical properties of trabecular bone, its contribution varies depending on the different loading types and test settings. To obtain the mechanical properties of trabecular bone comprehensively and accurately, the solid matrix (trabeculae) and fluid-like component (bone marrow) should be considered in parallel rather than tested separately.

**Supplementary Information:**

The online version contains supplementary material available at 10.1186/s12938-022-01051-1.

## Background

Trabecular bone is a hierarchical, spongy and porous structure, located mainly at the ends of the long bones (tibia, femur), within irregular shared bones (vertebrae, sacrum) and flat bones (skull, ribs) [1]. At the macrostructural scale, the structure consists of trabecular struts and plates that provide a stiff framework for cellular spaces, filled with bone marrow and cells in vivo [2].

Bone marrow, which is divided into red and yellow marrow, is a semi-solid soft substance located within the central cavity of long axial bone and the pore spaces of trabecular bone. Yellow (fatty) marrow is the main tissue filling trabecular bone in adult humans, and the composition of bone marrow varies greatly with anatomical site and age [3, 4]*.* Previous studies have reported that fat content increases in the process of bone loss such as osteoporosis or age-related osteopenia [5–8]. The characteristics of bone marrow have been investigated in previous publications. In a study by Davis et al. [9], the viscosity of bone marrow was measured. The data revealed that the specimens containing red components behaved like a non-Newtonian fluid in the range of 36–38 ℃, while yellow marrow without any red components behaved like a Newtonian fluid at 23 and 36 ℃. Jansen et al. [10] found that bone marrow is elastic and exhibits a large heterogeneity in both intra- and inter-specimens, with the effective elastic modulus at a physiological temperature ranging from 0.25 to 24.7 kPa. This raises a scientific question, does the presence of bone marrow, as part of the internal structure of trabecular bone, affect its mechanical properties? In other words, when accurately evaluating the mechanical properties of trabecular bone, is it possible to neglect the role of bone marrow?

Concerning this subject, previous studies have been conducted not only with in vitro experiments [11–14], but also with computer model simulations [15–17]. However, in practice, it is hard to quantify the contribution of bone marrow to the mechanical behavior of trabecular bone since it is highly reliant on the experimental setup and conditions [13, 14]. To date, there is a lack of comprehensive research on this topic. Several studies have investigated the mechanical properties of trabecular bone, focusing only on the solid matrix, and neglecting the role of bone marrow [18–20].

Therefore, a systematic review on this subject is necessary because it is valuable not only for obtaining more accurate in vitro experimental results, but also for building more accurate computer models (e.g., finite element models, FEMs) of trabecular bone. To be specific, this study aims to answer two scientific questions: (i) whether the presence of bone marrow would cause hydraulic stiffening of trabecular bone? (ii) What are the differences in the role of bone marrow under various mechanical test conditions of trabecular bone?

## Results

### Description of studies

**Fig. 1:**
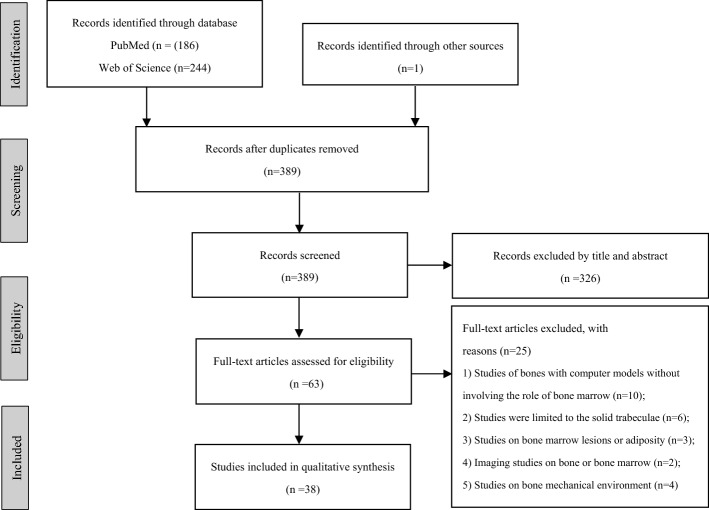
Flowchart displaying selection process

**Fig. 2:**
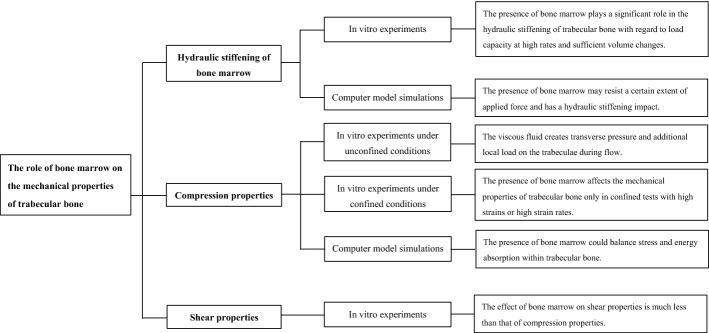
The flowchart on the main results of this review

Totally, 431 articles were found through PubMed (*n* = 186), Web of Science databases (*n* = 244), and other sources (*n* = 1). After removing duplicates, 389 studies were potentially eligible. Following the screening of titles, abstracts, and full-text articles, 38 articles were finally included in our study. Details are shown in Figs. 1, 2, and Additional file 1: Table S1.

### Risk of bias

All included articles (*n* = 38) were completed for quality assessment, and details are shown in Table 1. Most of the studies provided detailed information about the research framework. Thirty-seven clearly described the background, purpose, and objectives of the study in the abstract. No study included the sample size calculation, and only two studies addressed the missing data [14, 21]. Furthermore, the large heterogeneity and lack of randomized controlled trials made it impossible to carry out a meta-analysis.

**Table 1:** Quality assessment of potential risk bias

Author (year)	Checklist questions																Score
	Abstract	Introduction		Methodology						Results			Discussion			Others	
	1	2	3	4	5	6	7	8	9	10	11	12	13	14	15	16	
Swanson et al. 1966	★	★	★	★	0	0	0	0	0	★	0	★	★	0	★	0	8/16
Pugh et al. 1973	★	★	★	★	★	0	0	★	0	★	0	★	★	0	★	★	11/16
Cater et al. 1977	★	★	★	★	★	★	0	★	0	★	0	★	★	0	★	0	11/16
Kazarian et al. 1977	★	★	★	★	0	★	0	★	0	★	★	★	★	0	★	0	11/16
Bryant 1983	★	★	★	★	0	★	0	0	0	0	0	★	★	0	★	★	9/16
Simkin et al. 1985	★	★	★	★	0	★	0	★	0	★	★	★	★	0	★	★	12/16
Simon et al. 1985	★	★	★	★	★	★	0	★	0	0	0	★	★	0	★	0	10/16
Bayant 1988	★	★	★	★	0	★	0	0	0	★	★	★	★	0	★	0	10/16
Ochoa, Heck, et al. 1991	★	★	★	★	0	★	0	★	0	★	★	★	★	0	★	★	12/16
Ochoa, Sanders, et al. 1991	★	★	★	★	0	★	0	0	0	★	★	★	★	0	★	★	11/16
Linde et al. 1993	★	★	★	★	★	★	0	★	★	★	★	★	★	0	★	0	13/16
Deligianni et al. 1994	★	★	★	★	0	★	0	★	0	★	★	★	★	0	★	0	11/16
Ochoa et al. 1997	★	★	★	★	0	★	0	★	0	★	★	★	★	0	★	0	11/16
Mitton et al. 1997	★	★	★	★	★	★	0	★	0	★	★	★	★	★	★	★	14/16
Lim et al. 1998	★	★	★	0	0	★	0	★	0	0	0	★	★	0	★	0	8/16
Kasra et al. 1998	★	★	★	★	★	★	0	0	0	0	0	★	★	0	★	0	9/16
Hong et al. 1998	★	★	★	0	0	★	0	0	0	0	0	★	★	0	★	0	7/16
Nuccion et al. 2001	★	★	★	★	0	★	0	★	0	★	0	★	★	★	★	0	11/16
Hong et al. 2001	★	★	★	★	0	★	0	0	0	0	0	★	★	★	★	0	9/16
Ochia et al. 2006	★	★	★	★	★	★	0	★	0	★	0	★	★	★	★	0	12/16
Chaari et al. 2007	★	★	★	★	★	★	0	0	0	★	0	★	★	0	★	0	10/16
Kasra et al. 2007	★	★	★	★	★	★	0	★	0	★	★	★	★	0	★	0	12/16
Pilcher et al. 2010	★	★	★	★	★	★	0	0	0	★	0	★	★	0	★	0	10/16
Halgrin et al. 2012	★	★	★	★	★	★	0	★	0	★	★	★	★	0	★	0	12/16
Birmingham et al. 2013	★	★	★	0	0	★	0	★	0	0	0	★	★	★	★	★	10/16
Haider et al. 2013	★	★	★	★	★	★	0	★	0	★	0	★	★	0	★	★	12/16
Sandino et al. 2014	★	★	★	★	★	★	0	★	0	★	0	★	★	★	★	★	13/16
Ma et al. 2014	★	★	★	★	★	★	0	0	0	★	★	★	★	★	★	★	13/16
Metzger et al. 2015	★	★	★	★	★	★	0	★	0	★	0	★	★	0	★	★	12/16
Sandino et al. 2015	★	★	★	★	★	★	0	★	0	★	0	★	★	0	★	★	12/16
Chen et al. 2015	★	★	★	★	★	★	0	★	0	★	★	★	★	★	★	★	14/16
Laouira et al. 2015	0	★	★	★	0	0	0	0	0	★	0	★	★	0	★	0	7/16
Metzger et al. 2015	★	★	★	★	★	★	0	0	0	★	★	★	★	★	★	★	13/16
Metzger et al. 2016	★	★	★	★	★	★	0	0	0	★	0	★	★	0	★	★	11/16
Sandino et al. 2017	★	★	★	★	★	★	0	★	0	★	★	★	★	★	★	★	14/16
Pense et al. 2017	★	★	★	★	★	★	0	★	0	★	★	★	★	★	★	★	14/16
Bravo et al. 2019	★	★	★	★	0	★	0	0	★	★	★	★	★	0	★	0	11/16
Rabiatul et al. 2022	★	★	★	★	★	★	0	★	0	0	0	★	★	★	★	★	12/16
Number of fulfilling each item	37	38	38	35	22	35	0	24	2	30	17	38	38	12	38	18	
Percentage (%)	97.4	100	100	92.1	57.9	92.1	0	63.2	5.3	78.9	44.7	100	100	31.6	100	47.4	

The asterisk (★) means this item is fulfilled, and zero (0) means this item is not fulfilled. **1 = **Was the abstract summarized informatively? **2 = **Was the scientific background detailed? **3 = **Were the objectives clearly stated? **4 = **Did the study clearly describe the methodology? **5 = **Were the characteristics of the sample clearly described? **6–1 = **Were potential confounders clearly described (in vitro experiments)? **6–2 = **Were the model parameters explicitly stated (computer model simulation)? **7 = **Was the calculation of sample size reported? **8 = **Was the data analysis described in detail? **9 = **Did any missing data address in the study? **10 = **Did the study report the sample numbers? **11 = **Was the basic information of the sample described? **12 = **Were the main findings of the study clearly described? **13 = **Were the key results summarized? **14 = **Were the limitations of the study discussed? **15 = **Were the overall results explained in detail? **16 = **Were the sources of funding for the study described?

### Population characteristics

Table 2 summarizes the characteristics of the 38 studies. Among them, 27 articles addressed the subject regarding hydraulic stiffening effect of bone marrow, including in vitro experiments and computer model simulations [17, 22–47]. Nine publications investigated the role of bone marrow in compression tests under different settings, i.e., in vitro experiments under unconfined and confined conditions, computer model simulations [11–16, 21, 48, 49]. Relatively few controlled studies investigated the effect of bone marrow on the shear properties of trabecular bone [50, 51].

**Table 2:** Summary of study characteristics, specimen demographic details, and main findings or summaries

Experimental methods	Authors, year of publication	Journal of publication	Types of specimens	Numbers of specimens	Gender	Age	Anatomic sites	Main findings or summaries
FEM under compressive loading	Kasra et al. 1998	Journal of Biomechanical Engineering	A 3D-FEM of a rod-strut trabecular structure filled with bone marrow	–	–	–	Vertebrae trabecular bone	Hydraulic stiffening of trabecular bone due to the presence of bone marrow occurs when the applied loading rate is higher than the pore fluid diffusion rate
Dynamic compressive strain cycles	Ochoa et al. 1991	Journal of Biomechanical Engineering	Intact bone samples	26 limbs	–	About 2 years	Femoral heads (mature dogs)	A nature stiffening mechanism of viscous fluid may be present in intact trabecular bone, and the overall stiffness reflects the material properties of both the porous solid matrix and entrapped fluid
Static and dynamic tests	Swanson et al. 1966	Medical & biological engineering & computing	Intact bone samples	4	–	–	Femora	Trabecular bone is not hydraulically strengthened by the presence of bone marrow under moderate and physiological loading conditions such as normal walking
Small amplitude mechanical excitation	Pugh et al. 1973	Journal of Biomechanics	Bone slabs	20	–	–	Distal ends of the human left femora	The fluid in the intertrabecular spaces has no effect on the dynamic mechanical behavior
Stress-relaxation and cyclic loading	Metzger, Schwaner, et al. 2015	Journal of Biomechanics	Intact bone samples	9	–	6–8 months	Porcine femurs	The deformation of the pores under external forces would induce the motion of the fluid-like marrow, resulting in pressure and velocity gradients
Compressive loads tests	Kazarian et al. 1977	Spine	Intact bone samples	48	Male	26, 27, 32 and 38 years	Thoracic vertebrae	The mechanical behavior of the vertebral centrum was dependent on the strain rate. This was due to the hydraulic strengthening caused by the internal marrow at the higher strain rates
Split-Hopkinson bar testing and FEM	Pilcher, et al. 2010	Journal of Biomechanical Engineering	Cylindrical and cubic trabecular bone samples	34	–	–	Bovine tibia (proximal region)	The strength of trabecular bone significantly increases when testing at high strain rates in the range of 10^2^–10^3^ s^−1^
FEM under compressive loading	Rabiatul, et al. 2022	Journal of Materials: Design And Applications	A 3D-constructed FEM	–	–	–	Bovine femur bones	The fluid flow of bone marrow not only causes shear stress to the trabecular structure, but also has a certain hydraulic stiffening effect that occurs due to the presence of bone marrow
Porous elastic model	Hong et al. 2001	KSME International Journal	A cylindrical trabecular bone sample	1	–	–	Calf vertebral trabecular bone	The difference in pore pressure generation resulted in a significant increase in the predicted total stress at the fastest strain rate (10 per second)
A one-dimensional poro-elastic model	Hong et al. 1998	KSME International Journal	Trabecular bone	–	–	–	–	The total stress–strain behavior of trabecular bone was greatly affected by the applied strain rate. The incorporation of the fluid effect is recommended
FEM under compressive loading	Pense et al. 2017	Journal of the Mechanical Behavior Of Biomedical Materials	Cubic bone samples	7	Female	61 years	Human femoral neck	There is a significant strain rate-dependent poro-elastic hydraulic stiffening of bone tissue due to the fluid in trabecular bone pores
FEM under a realistic impact load	Haider et al. 2013	Journal of Biomechanics	Intact bone samples	1	–	–	Cadaveric femur	Hydraulic strengthening has little effect on whole bones in realistic fall conditions, as this load condition causes no volumetric strain
FEM under stress relaxation experiments	Sandino et al. 2015	Journal of the Mechanical Behavior Of Biomedical Materials	Cubes of trabecular bone (10 mm side-length)	33	–	64.35 (49–85)	Human distal tibia	The contribution of viscoelasticity (fluid flow-independent mechanism) to the mechanical response of the tissue is significantly greater than the contribution of the poro-elasticity (fluid flow-dependent mechanism)
FEM and in vitro permeability experiments	Sandino et al. 2014	Journal of Biomechanics	Cubes of trabecular bone (10 mm side-length)	23	–	–	Human distal tibia	The changes in the trabecular bone microarchitecture have an exponential relationship with permeability
FEM for mechanical stimuli of trabecular bone	Sandino et al. 2017	Journal of the Mechanical Behavior Of Biomedical Materials	Cubes of trabecular bone (10 mm side-length)	76	14 females, 6 males	68 ± 15 (49–95) years	Human distal tibia	With variations in the morphology of the trabecular bone, such as an increase of 30% porosity, there is a significant decrease in the mechanical stimuli of the tissue when subjected to constant strain
A one-dimensional poro-elastic model of trabecular bone	Lim et al. 1998	Journal of Musculoskeletal Research	–	–	–	–	–	Trabecular bone is poro-elastic and the fluid effect on the mechanical behavior at the continuum level is significant
Sinusoidal strain excitation	Ochoa et al. 1997	Journal of Biomechanical Engineering	Intact bone samples	38	–	About 2 years	Femoral heads (mature dogs)	Hydraulic effects measured in vivo accounted for an average of 19 percent of the load-bearing capabilities of the structure over the frequency range considered
A fluid structure interaction model	Birmingham et al. 2013	Annals of Biomechanical Engineering	–	–	–	–	–	Lower bone mass leads to an increase in the shear stress generated within the marrow, while a decrease in bone marrow viscosity reduces this generated shear stress
Fluid–structure interaction models (FEM under compressive loading)	Metzger, Kreipke, et al. 2015	Journal of Biomechanical Engineering	Cubes of trabecular bone (4 mm side-length)	2	–	–	Proximal and distal femoral neck	Compression of the bone caused a flow of the marrow within the trabecular pore space. The marrow moved slowly, with velocities lower than 0.1 mm/s, which was accompanied by concomitant shear stress and pressure gradient
Non-destructive impact loads	Bryant 1983	Journal of Biomechanics	Intact bone samples	–	–	–	Sheep tibiae	In non-destructive impact loading, low marrow pressures would provide only a trivial amount of hydraulic strengthening
Dynamic loading cycles	Ochoa et al. 1991	The Journal Of Rheumatology	Intact bone samples	24	–	At least 2 years	Hind limbs of dogs	The removal of fluid phase decreases the stiffness of trabecular bone in intact canine femoral head specimens (a more than 30% decrease in stiffness)
A load of physiologic magnitude	Nuccion et al. 2001	Orthopedics	Intact bone samples	24	–	–	Femora of mature dogs	Removal of the intraosseous fluid decrease the mechanical stiffness of canine trabecular bone (a 40% decrease in stiffness)
Hydrostatic pressure response	Simkin 1985	Journal of Biomechanics	Intact bone samples	20	4 female, 6 male	–	Arms and shoulders of dogs	When the trabecular bone deforms under unconstrained load, fluid will flow freely and will play no mechanical role as surrounding trabeculae are compressed. while enclosed fluid will directly transmit a portion of the loading rate
Hydraulic resistance testing	Ochia et al. 2006	Spine	Intact bone samples	21	–	67.4 ± 12.0 years, 73.3 ± 8.8 years	Human lumbar vertebrae (L3 and L4)	Marrow flow can be biphasic in nature at flow rates, and that marrow flow could potentially damage trabeculae and weaken the vertebral body during high-speed injury events
Compression testing	Deligianni et al.1994	Biorheology	Cylindrical and cubic specimens	22	10 females	55–70 years	Femoral heads	Under a step load and at strain rate, 10 min^−1^, the marrow can bear about 25% of the applied load
Solid and fluid constitutive models	Metzger et al. 2016	Journal of Biomechanics	Cubes (3 mm side-length)	2	–	–	Human femurs	The results differed substantially between elastic, hyperelastic, and viscoelastic constitutive models, even when using the same modulus
Unconfined compression test and FEM simulation	Halgrin et al. 2012	Journal of the Mechanical Behavior Of Biomedical Materials	Cubic trabecular bone samples	48	–	Less than 24 months	Bovine ribs	The bone marrow contributes to decrease the mechanical properties of trabecular bone, i.e., 26% for the elastic modulus, 38% for the maximum compressive stress, and 33% for the average stress
Unconfined compression test	Bravo et al. 2019	Biomedical Physics & Engineering Express	Cubic trabecular bone samples	60	–	About 5 months of age	Porcine femur bones	The samples extracted marrow and replaced with saline solution have higher mechanical properties
Unconfined compression test	Linde et al. 1993	Journal of Biomechanics	Cylindrical trabecular bone samples	74	Male	35 and 61 years	Human proximal tibial	Defatted trabecular bone specimens contribute to a 30% increase in stiffness and a 50% decrease in viscoelastic dissipation
Compression test and FEM	Chaari et al. 2007	International Journal Of Crashworthiness	Cylindrical trabecular bone samples	97	–	–	Beef ribs	The fluid influence is essentially observed in the last compression stage, when the sample’s strain is higher than 30%
Semi-constrained compression test	Carter et al. 1977	The Journal Of Bone And Joint Surgery	Cylindrical trabecular bone samples	124	–	–	Human tibial plateaus and bovine femoral condyles	The presence of marrow increased the strength, modulus, and energy absorption of specimens only at the highest strain rate of 10.0 per second
Dynamic compressive loading (FEM simulation)	Laouira et al. 2015	Computer Methods In Biomechanics And Biomedical Engineering	Cubic trabecular bone samples	1	–	–	Porcine femoral neck	Confined marrow plays a non-negligible role upon the mechanical properties of trabecular bone
Static and dynamic load	Bryant 1988	Journal of Engineering In Medicine	Human and sheep long bones	2	Male for human bones	50 years for human bones	Sheep tibia and human radius	Hydraulic strengthening and viscous resistance by the marrow can be negligible when there is little or no volume change, as well as no significant movement between the marrow and the adjacent trabecular bone
FEM under compressive loading	Chen et al. 2015	Annual International Conference Of The IEEE Engineering In Medicine And Biology Society	FEM of trabecular bone	4	Female	Post-menopausal	Human L3 lumbar spine	Trabecular models filled with marrow fat have less maximum stress (3–9%) and larger average stress in volume (9–56%) than that of models with only trabeculae
FEM under compressive loading	Ma et al. 2014	Annual International Conference of the IEEE Engineering In Medicine And Biology Society	FEM of trabecular bone	1	Female	63 years	Human L3 lumbar spine	The trabecular bone with marrow fat suffered larger apparent stress and compressive stress than the model with merely trabecular bone for unconfined compressive tests, i.e., 18.81% for the maximum compressive stress, 10.25% for the average stress
Creep test	Simon et al. 1985	Spine	FEM of trabecular bone	–	–	–	Rhesus monkey L2/L3 lumbar spine	The fluid phase included in the FEMs plays a significant role in the mechanical response of spinal motion segments
Shear test	Mitton et al. 1997	Medical Engineering & Physics	Cylindrical trabecular bone samples	43	Female	9 years (5.6–10.3 years)	Ewe vertebral trabecular bone (L1–L5)	The shear test performed in a bath at 37 ℃ reduced the strength from 32.5 to 37.3% compared with the test in “standard” test conditions. Friction was regarded as a non-negligible factor
Shear properties under torsional loading	Kasra et al. 2007	Journal of Biomechanics	Cylindrical trabecular bone samples	52	Female	6 months and 2 years	Merino sheep lumbar segments (L2–L3)	The presence of bone marrow did not affect trabecular bone shear strength and modulus

*3-D* three-dimensional, *FEM* finite element model

### Quality assessments questions

- Abstract
  - 1. Did the abstract provide an informative and balanced summary of what was done and what was found?
- Introduction
  - 2. Was the scientific background and rationale for the reported investigation explained?
  - 3. Were the objectives of the study clearly stated?
- Methodology
  - 4. Did the study clearly describe the methodology/protocol of studies which includes the setting, the sources, and sizes of samples included (the size is only applicable for standardized samples)?
  - 5. Did the characteristics of bone samples included in the study clearly described, including density, volume fraction, or porosity?
  - 6–1. Did the exposures, potential confounders, and allocation scheme for the samples have been clearly described (applicable to in vitro experiments)?
  - 6–2. Did the computer model provide a clear statement of the source of stimulation parameters with appropriate reasons or references (applicable to computer model simulation studies)?
  - 7. Was the calculation of study size/sample size reported?
  - 8. Were the statistical tests or data analysis methods used to access the main outcomes described in detail?
  - 9. Did any missing data address in the study?
- Results
  - 10. Did the number of samples included in the study have been reported in detail?
  - 11. Did the study indicate basic information about bone samples, including age, gender, species origin, and anatomical site (gender is only applicable to human bone samples)?
  - 12. Were the main findings of the study clearly described?
- Discussion
  - 13. Did the study summarize the key results with reference to study objectives?
  - 14. Were the limitations of the study discussed, taking into account sources of potential bias?
  - 15. Did the study interpret overall results considering objectives, the multiplicity of analyses and results from similar studies/relevant evidence?
- Other information
  - 16. Did the study state the source of funding or the role of funders for the present study?

### Hydraulic stiffening of bone marrow

Currently, both in vitro experiments and computer model simulations have been used to investigate the hydraulic stiffening of bone marrow. Comprehensive knowledge of this subject may enhance the understanding of important orthopedic problems.

### In vitro* experiments*

Theoretically, the deformation of the pores under external forces would induce the motion of the fluid-like marrow, resulting in pressure and velocity gradients [22, 40]. Because of diverse experimental setups and conditions, the impact of hydraulic stiffening and strengthening by bone marrow has proven contentious in practice [17, 23–27, 38, 42–46].

Under moderate and physiological loading conditions (i.e., normal walking), Swanson and Freeman [23] found that trabecular bone is not hydraulically strengthened by bone marrow. In line with this result, Pugh and co-workers [24] compared the mechanical properties of fresh wet and defatted bone specimens under the condition of small amplitude mechanical excitation (100 to 30000 Hz). According to the findings, the fluid in the intertrabecular spaces had no influence on the dynamic mechanical behavior. Bryant [28, 41] also found that hydraulic strengthening and viscous effects do not appear to occur in long bones subjected to non-destructive compression loads. They argued that when there is little or no volume change, as well as no significant movement between the marrow and the adjacent trabecular bone, the hydraulic strengthening and viscous resistance by the marrow may be insignificant.

Apart from the tests with small volume deformations, it was observed in a study by Kazarian et al. [25] that the mechanical behavior of the vertebral centrum was dependent on the strain rate. They explained that this was due to hydraulic strengthening caused by the internal marrow at the higher strain rates. In agreement with this result, the strength of trabecular bone increases significantly when testing at high strain rates in the range of 10^2^–10^3^ s^−1^, according to Pilcher et al. [29]. However, they did not consider the compressive loading of bone marrow to be an important effect. They explained that this is because the trabecular bone does not have enough time to occur due to the rapidly increasing applied stress, resulting in a different failure mechanism, i.e., higher failure stress and lower failure strain. Actually, not only for the solid structure of trabecular bone (trabeculae), but also for the internal fluid-like bone marrow, where the enclosed fluid would directly transmit a part of the load (25% of the applied load) when the trabecular bone deforms under external forces, according to the findings by Simkin et al. [44] and Deligianni et al. [46]. In addition, Ochoa et al. [26] investigated the influence of intraosseous fluid on the load capability of the intact canine femoral heads under in vitro conditions. The results revealed that intraosseous fluid within the femoral head provides a significant portion of the stiffness, up to 30% of the initial stress. Ochoa et al. [38] also performed the same experiment under in vivo conditions. In corresponds to similar results in vitro, intraosseous fluid within the femoral head provides a significant portion of the total stiffness, i.e., an average of 19% of the load-bearing capabilities. They explained that this difference (30% in vitro *vs*. 19% in vivo) is due to variations in temperature and rheological properties of bone marrow. The studies by Ochoa et al. [42] and Nuccion et al. [43] also support this view that the mechanical stiffness of the femoral head would be affected when the intraosseous fluid compartment is disrupted (a 33% reduction by Ochoa et al. and a 40% decrease by Nuccion et al.). Furthermore, under more high-speed loading condition (2500 mm/s), a study by Ochia et al. [45] indicated that the high fluid flow caused by bone marrow could result in the bending or breaking of trabeculae, which may damage trabeculae of the vertebral body.

### Computer model simulations

Apart from in vitro experiments, the computer model simulations such as FE, poro-elastic and viscoelastic models have also been utilized to investigate the hydraulic stiffening of trabecular bone caused by the presence of bone marrow [17, 27, 32–37, 39, 47]. A study by Metzger and co-workers [47] has reported that trabecular bone was simulated by different models (linear elastic, neo-Hookean, viscoelastic, and power-law fluid constitutive models) with significant variations in test results, in which the bone marrow as a fluid plays an important role. According to Sandino et al. [34], for trabecular bone, the fluid flow mechanism induced by bone marrow is a non-negligible role in the building of trabecular bone models and needs to be taken into account. Kasra et al. [17] also indicated that hydraulic stiffening occurs once the applied loading rate is higher than the diffusion rate of pore fluid. Consistent with this view, in other studies [30, 31], the enhancement of hydraulic stiffness was observed at faster loading strains. Pense and co-workers [32] also concluded that there is a significant strain-rate dependence of poro-elastic hydraulic stiffening in bone tissue due to the fluid in the trabecular bone pores. A study by Lim and co-workers [37] claimed that trabecular bone is poro-elastic and the fluid effect on the mechanical behavior at the continuum level is significant.

Investigation of hydraulic stiffening, not only in normal bones, but also provides a useful tool for understanding of the abnormal physiological in trabecular bone. For trabecular bone with aging or osteoporosis, the alternation of the microstructure also results in changes in its permeability, and in an exponential relationship [35]. There is no doubt that this alternation in permeability would affect the fluid flow and pore pressure generation significantly. This hypothesis was also confirmed in previous studies [36, 39]. According to Sandino et al. [36], when the porosity of trabecular bone increases by 30%, the average stress and strain in the bone tissue may reduce 50% and the fluid velocity in the marrow phase 88%. Also, Birmingham et al. [39] found that lower bone mass could increase the shear stress generated within the marrow, meanwhile, a decrease in bone marrow viscosity reduces the generated shear stress.

In addition to that, concerning the question of whether physiological loading (normal walking) causes hydraulic stiffening of the trabecular bone. The simulation results by Rabiatul et al. [27] indicated that, during normal walking loading, the presence of bone marrow may resist a certain extent of applied force, which caused the apparent stiffness of the trabecular structure. In contrast to this view, Haider et al. [33] used a patient-specific FEM to determine the effects of hydraulic strengthening on the structural response of the proximal femur under a realistic impact load. The results showed that the presence of bone marrow results in little hydraulic strengthening effect, i.e., 2% of the total hydraulic stress.

### Compression properties

Compression tests are used to determine how a material reacts when compressed by measuring basic parameters including elastic modulus, maximum compressive stress, average compressive stress, yield stress, toughness, etc. [13, 14]. Previous studies have investigated the role of bone marrow on the compression properties of trabecular bone, both in vitro experiments and computer model simulations.

### In vitro experiments under unconfined conditions

According to the findings by Halgrin et al. [13], under unconfined uniaxial compression test conditions, bone marrow contributes to a reduction in the mechanical properties of trabecular bone, i.e., 26% for elastic modulus, 38% for maximum compressive stress, and 33% for average stress. They explained that the viscous interstitial fluid creates transverse pressure and additional local load on the trabeculae during flow, increasing the transverse strain applied to the trabecula, causing the trabecular network to prematurely collapse. Consistent with Halgrin et al. [13], Bravo et al. [14] found that the specimens with the marrow removed and replaced with saline exhibited superior mechanical characteristics, i.e., 37% for elastic modulus, 48% for 0.2% yield stress, 39% for maximum compressive stress, 54% for strain at maximum stress, and 300% for toughness. They explained that during the unconfined compression testing, the vertical movement of bone marrow caused the fluid to expand horizontally, bending the trabeculae and decreasing the apparent strength. On another hand, during the deformation, the higher viscosity of bone marrow provides a greater barrier to fluid flow, leading to greater stress concertation alone the trabeculae and early breakdown of the trabecular structure. In an earlier study by Linde and co-workers [21], defatted trabecular bone specimens were shown to enhance stiffness by 30% while decreasing viscoelastic dissipation by 50%. They attributed the variation in mechanical properties of specimens with and without bone marrow to drying, and rehydration in saline for more than 3 h would diminish the discrepancies. Indeed, drying or dehydration of trabecular bone specimens can lead to changes in mechanical properties. However, to avoid dehydration, specimens in the experiments by Halgrin et al. [13] and Bravo et al. [14] were maintained in saline throughout all preparation processes. From our perspective, the presence of bone marrow, apart from the potential effect of dehydration, still plays a significant role in affecting the mechanical properties of trabecular bone during unconfined compression testing.

### In vitro experiments under confined conditions

In both confined and unconfined conditions, Chaari et al. [48] conducted quasi-static compression tests. According to the findings, there was no significant difference in elastic properties, but bone marrow may increase bone strength at higher strain (more than 30%). Moreover, according to the results by Cater and Hayes [11], at a very high strain rate (10.0 per second), the presence of bone marrow enhanced the strength, modulus, and energy absorption of trabecular bone specimens. This is due to the constricted viscous flow of bone marrow through the platen rather than the flow through the pores of the trabecular bone. Hence, the presence of bone marrow affects the mechanical properties of trabecular bone only in confined compression tests with high strains or high strain rates.

### Computer model simulations

A study by Simon et al. [49] revealed that the fluid phase contained in FEMs plays an important role in the mechanical response of spinal motion segments. Halgrin et al. [13] simulated the deformation of trabecular bone specimens using a FEM and reported that the fluid pressure caused by the bone marrow would reduce the maximum compressive stress. They claimed that the specimens with bone marrow had lower global axial stress and strain before collapse compared to specimens without bone marrow. The FEM simulations by Chen et al. [16] and Ma et al. [15] were both conducted under unconstrained conditions in the X and Y directions. The simulation results by Chen and co-workers [16] demonstrated that trabecular models stuffed with marrow fat have less maximum stress (3–9%) and larger average stress in volume (9–56%) than that of models with only trabeculae. They stated that the presence of marrow fat could improve the strength of trabecular bone by balancing stress and energy distribution. However, the FEM simulation results by Ma et al. [15] showed that the trabecular bone with marrow fat suffered larger apparent stress and compressive stress than the model with trabecular bone only, i.e., 18.81% for maximum compressive stress and 10.25% for average stress. They concluded that the bone marrow augmented the stress but balances the distribution of stress. The trabecular bone without marrow is more likely to fracture under mechanical loading due to unbalanced deformation. Moreover, a study by Laouira et al. [12] demonstrated that the confined marrow plays a non-negligible role in the mechanical properties of trabecular bone, i.e., 22.3% increase in maximum von Mises stress, 12.4% increase in maximum shear stress, 5.8% reduction in maximal strain. They explained that this is due to the increase in marrow pressure, which acts like a damper between the trabeculae, slowing down their deformation. Furthermore, the flow of bone marrow slows down the velocity of deformation of the solid trabeculae when an external force is applied.

### Shear properties

Specimens from trabecular bone are typically difficult to machine since the aged trabecular bone in humans is so fragile [50]. To date, few studies have investigated the effect of bone marrow on the shear properties of trabecular bone [51].

Nevertheless, some potential evidence can be found by summarizing the previous literature [28, 50]. Mitton et al. [50] measured the shear strength of trabecular bone specimens with and without physiological saline. The results showed that shear testing in a physiological saline bath at 37 ℃ reduced the strength from 32.5 to 37.5% compared to testing under “standard” conditions (at room temperature, 22–25 ℃, in the air). They claimed that friction may be a non-negligible factor. The yellow marrow, being a Newtonian fluid, has an approximately 10 times higher viscosity than that of water at 37 ℃ [28]. Hence, it is worth considering whether the presence of a highly viscous fluid would produce a non-negligible internal fraction on the shear properties of trabecular bone.

However, a controlled trial by Kasra and Grynpas [51] revealed a different view. The sheep lumbar vertebrae were used to test the shear properties of trabecular bone at different strain rates. According to the findings, the presence of bone marrow had no influence on shear modulus and strength at both low and high strain rates. In compression tests, the confined test condition and high loading rate cause the entrapped marrow to resist the compressive force. Contrarily, during torsion or shear loading, the bone volume of the tested specimen remains relatively unchanged and the stiffening effect caused by the friction between bone and marrow is much smaller [51]. It is reasonable to assume that the effect of bone marrow on shear properties is much less than that of compression properties. Certainly, this view needs to be verified by further research in the future.

## Discussion

The role of bone marrow on the mechanical properties of trabecular bone under different loading conditions was systematically reviewed. According to our results, the solid matrix (trabeculae) and fluid-like component (bone marrow) should be considered in parallel rather than tested separately. Cleaning or replacing the marrow with other solutions (e.g., physiological saline) in the in-trabecular space would change the mechanical behavior of trabecular bone. Undoubtedly, this information is important for the prevention and treatment of degenerative bone diseases (e.g., osteoporosis), and fragility fracture, as well as building more accurate in vitro models of trabecula bone.

### Biomechanical characteristics of bone marrow

Bone marrow is generally divided into two types, red marrow, which has a hematopoietic function, and yellow marrow, which is rich in fat. In healthy adults above the age of 25 years, yellow marrow accounts for a major part of the bone marrow (70% of adult bone marrow volume) [52]. On the contrary, red marrow is predominant in early childhood. However, the difficult harvesting of red marrow limits the ability to isolate and test its mechanical characteristics by conventional approaches [10]. As a result, more biomechanical studies on bone marrow have focused on yellow marrow. For instance, Jansen et al. [10] used three different techniques (rheology, indentation, and cavitation) to evaluate the mechanics of intact yellow porcine bone marrow. The results indicated that bone marrow is elastic, with an effective Young’s modulus of 0.25–24.7 kPa at physiological temperature; moreover, there is a high degree of heterogeneity in both intra- and inter-specimens. Actually, in vivo, the composition (adipose tissue fraction) [8, 53] and mechanical characteristics (e.g., viscosity, dynamic moduli) [10, 28] of yellow marrow present dynamic alternations with age and temperature. In turn, the alternations in the composition and mechanical characteristics may further affect the role of bone marrow on the mechanical properties of trabecular bone. A study by Fazeli et al. [52] concluded that an inverse association between marrow adipose tissue and measures of bone strength.

In addition, the heterogeneity of bone marrow and surrounding cortical bone is also a challenge for researchers interested in conducting mechanical studies. The structure and histology of bone marrow are governed by numerous variables related to specimen collection and processing [3]. Also, previous studies used bone marrow extracted from the medullary cavity for histological and mechanical properties, but this method is destructive and there is a gap with the properties of intact bone marrow [10, 28, 54, 55]. Taken together, the mechanical characteristics demonstrated by bone marrow at the organ level are a complex and dynamic behavior. It is still a challenging issue to investigate the mechanical characteristics of the bone marrow itself comprehensively and accurately.

### Hydraulic characteristics of fluid–solid interaction

The hydraulic nature of this fluid–solid interaction has a potential impact on the mechanics of trabecular bone, particularly in intact bone where the boundary condition has not been disrupted [25, 26, 49]. As far as we know, no previous systematic review has been carried out upon the effect of bone marrow on the mechanical properties of trabecular bone. In practice, understanding the fluid flow, changes, and hydraulic stiffening mechanism of bone marrow is of potential clinical significance. For example, the impact of viscous constituents during mechanical loading is referred to as hydraulic stiffening of trabecular bone, which is a more realistic simulation for physiological falls [32]. Not only that, hydraulic resistance and permeability are also believed to be potentially associated with high-speed spinal injuries such as burst fractures [29, 56]. Currently, based on the above findings [17, 24–26, 28, 30–32], we may reasonably conclude that the hydraulic stiffening and strengthening of trabecular bone associated with bone marrow is minimal or even neglectable at small strains (i.e., non-destructive loading). The presence of bone marrow, however, plays a significant role in the hydraulic stiffening of trabecular bone with regard to load capacity at high strain rates and sufficient volume changes. In vivo, the overall stiffness of trabecular bone is actually a combination of the material properties of the porous solid substrate and enclosed fluid.

### The effect of bone marrow on compressive loading

Experiments addressing the mechanical properties of trabecular bone are often conducted on the cadaveric bone to reflect in vivo performance. To date, the majority of investigations have studied the effect of bone marrow on compressive loading, in both unconfined and confined situations [11, 13, 14, 57]. Under the unconfined condition, bone marrow can flow freely when subjected to compressive loading. Viscous bone marrow creates transverse pressure and extra local stress on the trabeculae during flow, which can cause a reduction in the mechanical properties of trabecular bone [13, 14]. However, the FEM simulation by Chen et al. [16] claimed that marrow fat can balance the load distribution of bone tissue, potentially reducing deformation under compressive stresses. Although the application of FEM can mitigate the limitations of existing in vitro experiments by taking advantage of reproducibility and repeatability. The drawbacks of FEM need to be carefully considered, i.e., bone marrow is simplified [16], bone matrix and marrow are regarded as solid homogeneous materials with consistent Young’s modulus [15]. So, the simulation results by FEMs are able to provide us with trends and references but cannot replace in vitro experiments. In contrast to the unconfined condition, fluid flow is prevented in the confined test. Based on the studies mentioned above [11, 12, 48], it is reasonable to conclude that bone marrow contributes to the mechanical properties of trabecular bone, especially at high strain rates and sufficient volume changes.

### The effect of bone marrow on other loads

Regarding the role of bone marrow on other mechanical loads, such as shear, tensile, and bending tests, few controlled studies have investigated this subject. As far as we know, only the study by Kasra and Grynpas [51] directly investigated the effect of bone marrow on the shear properties of trabecular bone by in vitro experiments. Because there is minimal change in bone volume and any stiffening impact is generated by considerably lower frictional forces between bone matrix and bone marrow, the presence of bone marrow had no significant influence on the shear modulus and strength of trabecular bone. Nevertheless, this view still needs to be validated by further studies in the future.

### Limitations

This systematic review has several limitations. First, not all studies were summarized in our review, which is a limitation of all systematic reviews. To overcome this problem, the “similar articles” option of PubMed and references of primary articles and reviews were used to further expand the search. Second, most studies related to the effect of bone marrow are on compressive loading, lacking direct compared studies on shear and other tests. However, reasonable assumptions have been proposed based on other relevant evidence from previous research. We believe that this review paper could shed new light on the knowledge gained so far, the drawbacks of existing literature, and future directions.

## Conclusion

To address the mechanical properties of trabecular bone, the role of interstitial fluid should be included in the analyses. In the confined or intact bone compression tests, hydraulic stiffening and strengthening of trabecular bone are associated with the presence of bone marrow, especially at high strain rates and sufficient volume changes. While in the unconfined compression tests, the free flow of viscous marrow under external forces induces the transverse pressure and extra local loading on the trabeculae. Bone marrow has a much smaller effect on shear properties than on compression properties since the bone volume of the tested specimen remains relatively unchanged. In shear and other tests, the potential role of bone marrow needs to be investigated by further studies in the future.

## Methods

The PRISMA (Preferred Reporting Items for Systematic review and Mata analysis) guidelines [58] were used to conduct a systematic review of the literature to find all relevant studies. Ethical approval was not required since this review did not include the processing of individual patient data.

### Information source

Using PubMed and Web of Science databases, a comprehensive review of the literature published up to June 2022 related to the role of bone marrow on the mechanical properties of trabecular bone was undertaken. The references of primary articles and reviews were checked to avoid missing relevant papers. The “similar article” option of PubMed was also used to further expand the search.

### Search strategy

Two reviews (F.W. and L.Z.) conducted an independent search. The following keywords were used to search from PubMed and Web of Science databases. In PubMed, the terms were performed for searching: (1) “(marrow [Title])” AND “(cancellous OR trabecular OR spongy)” AND “(mechanical OR compress^*^ OR tens^*^ OR shear^*^ OR bending)”; (2) “(mechanical stimuli) OR (permeability) OR (poro-viscoelastic)” AND “(trabecular bone [Title])” OR “(cancellous bone [Title])” OR “(spongy bone [Title])” AND “(finite element [Title])”; (3) “(hydraulic [Title]) OR (boundary conditions [Title])” AND “(trabecular bone) OR (cancellous bone) OR (spongy bone) OR (fracture strength) [Title])”. In Web of Science, the terms: (1) “(marrow) AND (trabecular OR cancellous OR spongy) [Title] AND (mechanical OR compress^*^ OR tens^*^ OR shear^*^ OR bending)”; (2) “(mechanical stimuli) OR (permeability) OR (poro-viscoelastic)” AND “(trabecular bone [Title])” OR “(cancellous bone [Title])” OR “(spongy bone [Title])” AND “(finite element [Title])”; (3) “(hydraulic [Title]) OR (boundary conditions [Title])” AND “(trabecular bone) OR (cancellous bone) OR (spongy bone) OR (fracture strength) [Title])” were used for literature search. The journal language was limited to English**.** In the Web of Science database, document types were set to “articles”. Following the removal of duplicates, reviewers scanned the search results by titles and abstracts. After identifying potentially relevant publications, full-text articles were reviewed and downloaded in accordance with the inclusion and exclusion criteria. Any disagreements between the two authors were referred to a third independent author to be discussed. The detailed search strategy is shown in Fig. 1.

### Inclusion and exclusion criteria

The inclusion criteria for this study were as follows: (a) in vitro mechanical tests of trabecular bone related to bone marrow; (b) studies on computer model simulations of trabecular bone associated with bone marrow; (c) studies on hydraulic stiffening of bone marrow. The exclusion criteria were: (a) non-English and full-text articles are unavailable; (b) studies of bones with computer models without involving the role of bone marrow; (c) studies were limited to the solid trabeculae; (d) studies on bone marrow lesion or adiposity; (e) imaging studies on bone and bone marrow; (f) studies on the bone mechanical environment.

### Data extraction and analysis

Data were extracted and recorded separately by two authors (F.W. and L.Z.) using spreadsheet software (Excel for Mac 2016, version 16.2.9, Microsoft, Redmond, WA, USA). Experimental methods, authors and year of publication, journal of publication, types and numbers of specimens, gender and age of specimens, anatomical sites, main findings or summaries were all presented.

### Quality assessment

The STROBE (Strengthening the Reporting of Observational Studies in Epidemiology) criteria were used to assess the risk of bias for the studies included in this review [59]. Of these, 16 items were selected to identify potential sources of bias related to the scope and objectives of our review for reporting, referring to a published article [60]. The checklist includes 6 components: abstract (item 1), introduction (items 2–3), methodology (items 4–9), results (items 10–12), discussion (items 13–15), and other information (item 16). All included articles were evaluated independently by two authors (F.W., and L.Z.). Disagreements were documented by discussion.

## Supplementary Information

**Additional file 1: Table S1.** List of excluded literature that did not meet the inclusion criteria.

## Data Availability

The data that support the findings of this study are available from the corresponding author upon reasonable request.

## Abbreviations

FEM: Finite element model
PRISMA: Preferred reporting items for systematic review and meta-analysis
STROBE: Strengthening the reporting of observational studies in Epidemiology
