# Supplementary material for: The role of bone marrow on the mechanical properties of trabecular bone: a systematic review
Source: Biomed Eng Online. 2022 Nov 23;21:80. doi: 10.1186/s12938-022-01051-1 (PMC9686043; doi:10.1186/s12938-022-01051-1)
Supplement: Supplementary file 1 — Additional file 1: Table S1. List of excluded literature that did not meet the inclusion criteria. [file 12938_2022_1051_MOESM1_ESM.docx]

Additional file 1: Table S1. List of excluded literature that did not meet the inclusion criteria.

| **Authors, year** | **Title** | **Exclusion reasons** |
| --- | --- | --- |
| Panyasantisuk J et al. 2016 | Effect of boundary conditions on yield properties of human femoral trabecular bone. | Studies of bones with computer models without involving the role of bone marrow. |
| Talukdar RG et al.2021 | Numerical analysis of the mechanical behavior of intact and implanted lumbar functional spinal units: Effects of loading and boundary conditions |  |
| Pahr DH et al. 2008 | Influence of boundary conditions on computed apparent elastic properties of cancellous bone |  |
| Guha I et al. 2022 | Finite element analysis of trabecular bone microstructure using CT imaging and continuum mechanical modeling. |  |
| Wang C et al. 2009 | Scale and boundary conditions effects on the apparent elastic moduli of trabecular bone modeled as a periodic cellular solid |  |
| Edwards WB et al. 2011 | Simulating distal radius fracture strength using biomechanical tests: a modeling study examining the influence of boundary conditions. |  |
| Bessho M et al. 2009 | Prediction of proximal femur strength using a CT-based nonlinear finite element method: differences in predicted fracture load and site with changing load and boundary conditions. |  |
| Bevill G et al. 2009 | The influence of boundary conditions and loading mode on high-resolution finite element-computed trabecular tissue properties |  |
| Yerby SA et al. 1998 | The effect of boundary conditions on experimentally measured trabecular strain in the thoracic spine. |  |
| Yeni YN et al. 2001 | Finite element calculated uniaxial apparent stiffness is a consistent predictor of uniaxial apparent strength in human vertebral cancellous bone tested with different boundary conditions. |  |
| Ding M et al. 2002 | Age-related variations in the microstructure of human tibial cancellous bone | Studies were limited to the solid trabeculae without involving the role of bone marrow. |
| Yoo A et al.2005 | Couple-stress moduli of a trabecular bone idealized as a 3D periodic cellular network. |  |
| Goda I et al. 2015 | Identification of couple-stress moduli of vertebral trabecular bone based on the 3D internal architectures |  |
| Synek A et al. 2016 | Biomechanical Testing of Distal Radius Fracture Treatments: Boundary Conditions Significantly Affect the Outcome of In Vitro Experiments. |  |
| Dong XN et al. 2004 | Effects of end boundary conditions and specimen geometry on the viscoelastic properties of cancellous bone measured by dynamic mechanical analysis |  |
| Syahrom A et al. 2011 | Mechanical and microarchitectural analyses of cancellous bone through experiment and computer simulation. |  |
| Matheny JB et al. 2017 | An in vivo model of a mechanically induced bone marrow lesion. | Studies on bone marrow lesions or adiposity without involving the study of the mechanical properties. |
| Scheller EL et al. 2016 | Changes in Skeletal Integrity and Marrow Adiposity during High-Fat Diet and after Weight Loss |  |
| Woods GN et al. 2020 | Greater Bone Marrow Adiposity Predicts Bone Loss in Older Women. |  |
| Rossi C et al. 2005 | DTI of trabecular bone marrow. | Imaging studies on bone or bone marrow |
| Lammentausta E et al.2006 | Prediction of mechanical properties of trabecular bone using quantitative MRI. |  |
| Coughlin TR et al. 2012 | Fluid shear stress in trabecular bone marrow due to low-magnitude high-frequency vibration | Studies on bone mechanical environment |
| Li T et al. 2020 | Fluid-solid coupling numerical simulation of trabecular bone under cyclic loading in different directions. |  |
| Fyhrie DP, et al. 1999 | Cancellous bone biomechanics |  |
| Webster D et al. 2014 | Strain energy density gradients in bone marrow predict osteoblast and osteoclast activity: a finite element study. |  |
